# Frequency or Amplitude?—Rheo-Electrical Characterization of Carbon Nanoparticle Filled Epoxy Systems

**DOI:** 10.3390/polym10090999

**Published:** 2018-09-07

**Authors:** Hauke Meeuw, Valea Kim Wisniewski, Bodo Fiedler

**Affiliations:** Institute of Polymer and Composites, Hamburg University of Technology (TUHH), Denickestr. 15, 20173 Hamburg, Germany; valea.wisniewski@tuhh.de (V.K.W.); fiedler@tuhh.de (B.F.)

**Keywords:** rheology, nanocomposites, agglomeration, dispersion

## Abstract

Dispersion of carbon nanoparticles in epoxy resin is the key factor to adjust the resulting electrical and mechanical properties of the nanocomposite. A profound understanding of the driving forces of standard methods like ultrasonic and mechanical dispersion is necessary. To derive the impact of applied frequency and strain on the resulting dispersion of multi-walled carbon nanotube (MWCNT)-filled epoxy resin, this work addresses the strain and frequency dependency of oscillatory shear flow-induced network changes. Strain- and frequency-sweeps were performed for a wide parameter set with in-line measurement of electrical DC resistance to monitor changes in the MWCNT network. Changes in electrical resistance reveal destruction and formation of the MWCNT network. A fundamental novel finding is the governing dependency of changes in the electrical network on applied shear amplitude. The applied frequency barely induces network changes. Applied shear rates do not correlate with particular network states.

## 1. Introduction

The addition of filler particles into a polymer matrix enables the adjustment of its physical properties. For instance, the addition of carbon-based nanoparticles leads to the electrical conductivity of the intrinsic isolating polymer. This is considered to be the most promising application [1]. Furthermore, the addition of this filler type results in an enhancement of mechanical properties, especially the fracture toughness [2]. These properties depend strongly on the distribution of the incorporated particles and the resulting interconnected network. The raw materials usually come along in an agglomerated state. To break down these agglomerates, high energy input into the system is necessary. This is realized by a dispersion process. Available processes are ultrasonication, high speed stirring, ball milling or three-roll-milling (TRM) [2,3]. Ultrasonication realizes the energy input by a high frequency oscillation vibration. This creates voids in the liquid, which collapse violently, releasing energy. This phenomenon is called cavitation [4]. Particles, accelerated up to 1000 km/h, collide with each other, and the agglomerates separate. High speed stirring and ball milling utilize the steady movement of either a stirring rod or the rotational movement of rigid balls. TRM drags the material into a micrometer gap between counter rotating rollers to generate shear forces. Here, the generated shear stress τ depends directly on the liquid viscosity η and shear rate $\dot{\gamma }$.

(1) $$\tau =\eta \dot{\gamma }$$

For instance, Couette geometries offer strain rates of 4000 s^−1^. Three-roll-milling and ultrasonication generate high shear rates of 10^5^ s^−1^ [5] and 10^9^ s^−1^ [6], respectively. Hence, they lead to the best dispersion grade [7]. The dispersion of CNT into polymers was intensively investigated. Schulz et al. observed superior dispersion results for TRM [8]. Nevertheless, high shear mixing and sonication raise unsolved issues, like the fact that the continuous TRM process leads to better dispersion grades than dispersion by way of higher shear rate oscillatory ultrasonication [1]. Ultrasonication leads to the fracture of CNT, which results in lower maximum conductivity at higher filler loading [9]. TRM does not lead to the fracture of CNT according to Gojny et al. [2]. Another disadvantage of ultrasonication is the fast heating up of the material and very local energy input [10]. TRM offers local high shear forces in the gap and a short dwell time, resulting in moderate heat input, as well as controllable and mild dispersion [7]. To address the different dispersion results by steady and oscillating dispersion methods, a profound look at the rheological properties is necessary to identify the shear-induced agglomeration and separation mechanisms in suspensions. The next section introduces the theory of rheology and gives an overview of the research that has been done regarding rheological characterization of particle-filled polymers.

## 2. Theoretical Background and State of the Art

A material between two parallel plates, which have differential parallel moving velocities, is exposed to shear. The resulting shear stress τ in the material depends on the applied shear deformation γ and shear modulus *G*.

(2) τ=Gγ

The viscosity of the material describes its resistance to deform under shear stress, results from inner friction of the material and is described by Equation (1). For a rheometer consisting of two rotational plates with a radius R, spacing h and rotational angle θ, the shear deformation γ is given by:

(3) $$\gamma =\frac{R}{h}\;\cdot \;\theta$$

By derivation, $\dot{\gamma }$ is defined as:

(4) $$\dot{\gamma }=\frac{R}{h}\;\cdot \;\frac{d\theta }{dt}$$

For an oscillatory excitation, the shear deformation with the angular frequency ω and shear amplitude $\gamma_{0}$ is given by:

(5) $$\gamma \left(\omega ,t\right)=\gamma_{0}sin\left(\omega t\right)$$

and consequently, the shear rate $\dot{\gamma }$ is given by:

(6) $$\dot{\gamma }\left(\omega ,t\right)=\gamma_{0}\;\cdot \;\omega \;\cdot \;cos\left(\omega t\right)$$

Due to the non-ideal viscous behavior of most materials, the resulting shear stress τ is out of phase, and the complex shear modulus $G^{*}$ consists of an elastic storage modulus $G^{\prime }$ and viscous loss modulus $G^{\prime \prime }$.

(7) $$\tau \left(\omega ,t\right)=G^{\prime }\;\cdot \;\gamma_{0}\;\cdot \;sin\left(\omega t\right)+G^{\prime \prime }\;\cdot \;\gamma_{0}\;\cdot \;cos\left(\omega t\right)$$

(8) $$|G^{*}|=\sqrt{G^{\prime 2}+G^{\prime \prime 2}}$$

For a fully-elastic material, $G^{*}$ would be equal to $G^{\prime }$ and for a viscous fluid equal to $G^{\prime \prime }$. The phase difference δ can be derived from the following equation:

(9) $$tan\left(\delta \right)=\frac{G^{\prime \prime }}{G^{\prime }}$$

$G^{\prime }>G^{\prime \prime }$ solid-like;   $G^{\prime \prime }>G^{\prime }$ fluid-like;   tan(δ)=1 fluid solid cross over

This results in a complex viscosity $\eta^{*}$, which is defined as:

(10) $$\eta^{*}=\frac{G^{*}}{\omega }$$

Cox and Merz postulated that $\eta \left(\dot{\gamma }\right)=\eta^{*}\left(\omega \right)$ for simple structured polymers [11].

**Figure 1:**
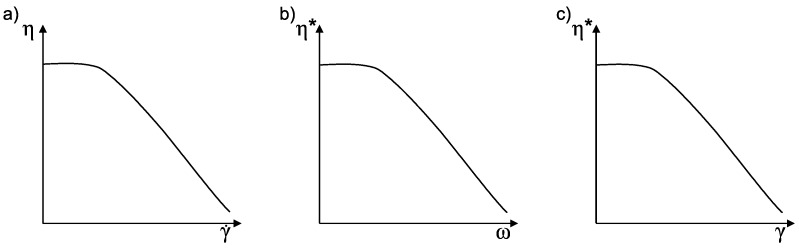
Overview of reported rheological behaviors of MWCNT/polymer suspensions: shear-thinning for: (**a**) steady flow with increasing shear rate (rate-sweep); (**b**) oscillatory flow with increasing angular frequency (frequency-sweep); (**c**) oscillatory flow with increasing strain amplitude (amplitude-sweep).

According to Equation (4), the shear rate in steady shear flow increases with increasing angular velocity. For oscillatory flow, according to Equation (6), $\dot{\gamma }$ increases with higher angular frequency ω or strain amplitude $\gamma_{0}$. The shear rate-dependent network changes of particle-filled polymers were investigated intensively for steady shear flow, and the structure analysis by rheology for MWCNT polymer suspensions was studied widely. Three types of material behavior can be observed for carbon nanoparticle-filled polymers. Figure 1 gives a schematic overview of the rheological material behaviors reported in the literature.

Most rheological results in the literature are reported for filled thermoplastic polymers. Figure 1a describes a pseudoplastic material behavior under increasing steady shear rate. The viscosity is shear rate independent until a yield point, and shear-thinning occurs subsequently [8,12]. In low viscosity systems like unsaturated polyesters, shear-thinning occurs without a yield point [13]. A parallel measurement of the electrical DC properties reveals deeper structural changes of carbon nanoparticle-filled polymers. This method was firstly reported by Skipia et al. in 2009 [14]. Low steady shear rates lead to carbon nanoparticle agglomeration in epoxy resins (EP) [14,15,16,17,18] and thermoplastic PC [19,20]; whereas high shear rates break down agglomerates and therefore the conductive network [16,21]. Independent of the initial dispersion state, the final resulting electrical particle network is directly connected to the applied steady shear rate [20,22,23]. An increase in viscosity accompanies increased electrical conductivity due to the build-up of the particle network [24]. The shear-thinning and change of the network is due to the rearrangement of particles and the orientation of polymer chains initiated by the shear rate [18,24,25]. Beside the steady shear results, there are data available for oscillatory frequency and strain-sweeps. Oscillatory experiments are most suitable to assess the internal structure of a material.

Figure 1b schematically shows the dependency of the complex viscosity of carbon nanoparticle-filled polymers on angular frequency. With increasing angular frequency, the complex viscosity decreases due to increasing shear rate (Equation (6)). Shenoy et al. postulated that the results from dynamic experiments would not lead to any new conclusions [12]. Nevertheless, there are few experimental data available for this strong shear-thinning behavior in EP [26,27], as well as for thermoplastic matrices [28,29,30]. The storage modulus determined in oscillatory experiments and electrical conductivity are in good accordance. A high storage modulus comes along with a pronounced three-dimensional particle network at low frequencies [20,31,32]. Most of the studies focused on the dependency of the filler content on either the electrical or rheological properties. An increasing filler content leads to a higher storage modulus, yield stress and electrical conductivity with pronounced independency of $G^{\prime }$ for low frequencies independently of the used polymer [13,18,27,29,33,34,35,36]. Chapartegui et al. concluded that higher angular velocities lead to more destruction of the filler network [18].

The dependency of complex viscosity on applied shear strain amplitude is schematically shown in Figure 1c. A pronounced shear-thinning is observed in this case, as well [37,38]. The impact of oscillatory strain on particle networks, in the case of viscosity, is higher for low molecular weight polymers [39]. Richter et al. showed by experiments and a theoretical approach that shear amplitude leads to a change of particle clusters [40]. An increase of $G^{\prime }$ and conductivity at small deformations is reported by Handge et al. [41] and a decrease of conductivity with accompanying shear-thinning for high strains by Zeiler et al. [42].

The identification of the mechanisms for CNT/polymer is complex, because three types of networks occur [25]:

I polymer network, by entanglements of chains
II carbon nanotube network, by interconnected CNT
III combined nanotube polymer network

Especially for high molecular weight thermoplastic materials, which show a distinct non-Newtonian visco-elastic behavior, the mechanisms of the network changes strongly interfere [43]. By using low molecular weight polymers, a reduction to only the filler network is possible [18]. Summarizing, an increase in shear rate leads to a structural change of the composite material, independent of increasing angular velocity in steady shear or increasing shear amplitude or angular frequency in oscillatory shear. The mechanisms behind the impact of strain amplitude and angular frequency on the particle network remain unresolved. There are no results regarding oscillatory rheo-electrical characterization of MWCNT/EP composites reported yet to answer this. Dispersion of nanoparticles in polymer matrices is crucial in many areas, the incomplete understanding of fundamental mechanisms of particle network formation is therefore an important field to study [44]. This paper focuses on the success of TRM dispersion, which is not based on shear rate only and explains the agglomeration of particles while processing by assessing the changes in network morphology by characterizing electrical properties under shear. The novelty of this research study is the approach to differentiate shear rate-induced structural changes either by increasing frequency or shear amplitude.

## 3. Materials and Methods

As the polymer matrix, the low molecular weight bisphenol-A-diglycidyl-ether epoxy resin Epikote 828LVEL, supplied by Hexion, Duisburg, Germany, was chosen. Its viscosity is 10.0–12.0 Pas at 25 ${}^{\circ }$C. As filler particles, NC7000 multi-walled carbon nanotubes, supplied by Nanocyl, Sambreville, Belgium, were used. The particles were dispersed into the matrix via a three-roll-mill 80E Plus, provided by EXAKT Advance Technologies, Norderstedt, Germany. The process parameters were adapted from a previous study [44]. An adjustment of the roller speed was necessary to match the resulting shear rates with a reduced diameter of 80 mm. Table 1 lists the process parameter of the 7-step process.

Table 2 summarizes the physical properties of the MWCNT used.

**Figure 2:**
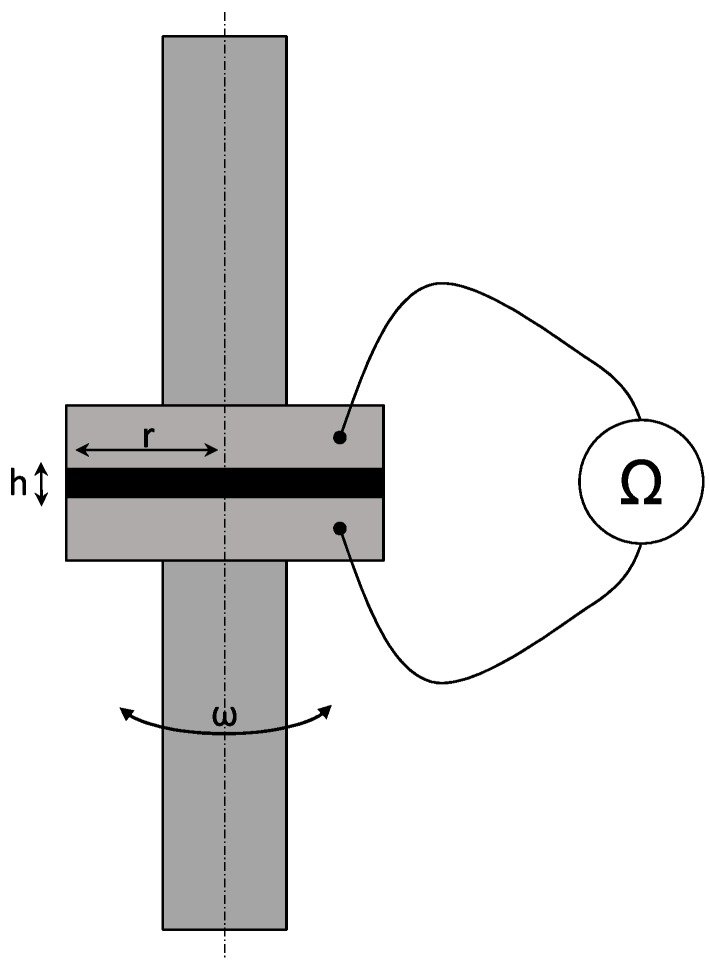
Rheo-electrical test setup.

In a previous study, a percolation threshold of 0.3 wt.% was observed for NC7000 dispersed in epoxy resin [45]. Hence, a filler loading of 0.5 wt.% was chosen to guarantee a solid-like behavior. To monitor the shear rate-induced changes of the particle network, a rheo-electrical characterization was carried out. Therefore, an ARES RDA-III 28, TA Instruments, New Castle, PA, USA, rheometer was enabled to measure, parallel to the rheological properties, the electrical DC resistance by utilizing a 2601A system source meter from Keithley, Cleveland, OH, USA. The electrical DC resistance was measured with 1 V from between the two plates. To characterize the morphological changes, strain controlled frequency-sweeps at different strain amplitudes and strain-sweeps with different angular frequencies were carried out. The frequencies for strain-sweeps varied from 0.01 Hz– 80 Hz and the strain amplitude levels for frequency-sweeps from 0.1–20%. Frequency-sweeps were carried out for a frequency range from 0.01 Hz– 80 Hz and strain-sweeps from 0.1–100%. To verify the Newtonian behavior of the neat epoxy resin, strain- and frequency-sweeps were performed in the same range. The test setup was a plate-plate configuration with a radius *R* of 40 mm and a spacing *h* of 500 μm. The chosen gap ensures that big agglomerates are not trapped between both plates. Figure 2 visualizes the test setup. The time-dependent relaxation of the material after pre-shearing due to the dispersion process was investigated by impedance spectroscopy and a long time oscillation test with parallel measurement of the DC resistance. The test frequency was 5 Hz, and the strain amplitude was 0.5%. An HP 4284A precision LCR meter coupled over a GBIBinterface into LabVIEW was used for impedance measurements. A detailed description of the impedance measurements can be found in [44].

## 4. Results and Discussion

**Figure 3:**
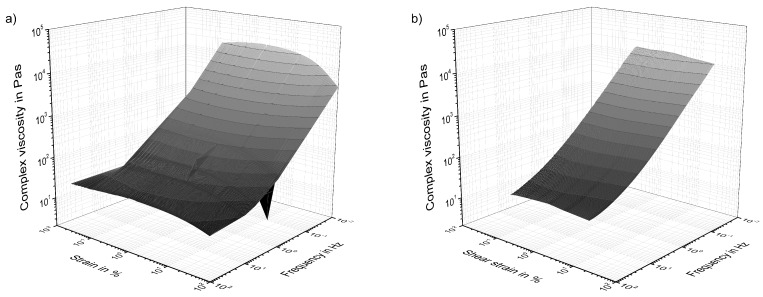
3D visualization of complex viscosity development for 0.5 wt.% NC7000 dispersed in Epikote 828LVEL gathered from (**a**) strain-sweeps at different frequencies and (**b**) frequency-sweeps at different strain levels.

As reported in the literature, the strain- and frequency-dependent complex viscosity of 0.5 wt.% NC7000/EP-composite shows a shear-thinning behavior. Figure 3a shows the behavior of the material resulting from strain-sweeps for different measurement frequencies. Figure 3b shows the behavior of the material from frequency-sweeps at different strain levels. Both experiments result in the same plot. Each rheological state is defined by a frequency and a shear strain. The complex viscosity of the material is more sensitive to frequency than to applied shear strain. It decreases, with an initial plateau, for increasing frequencies and reaches a lower plateau. For strain-sweeps, the complex viscosity shows a pronounced initial plateau and starts to decrease at a yield point. The explanation of this material behavior is given in the following sections.

### 4.1. Rheological Properties of Neat Resin

**Figure 4:**
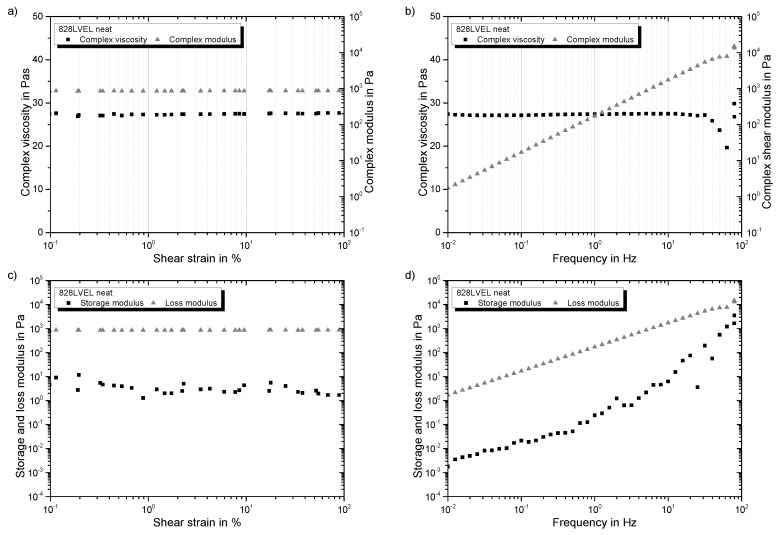
Oscillatory rheology results of 0.5 wt.% NC7000 in Epikote 828LVEL: (**a**) dependency of complex viscosity and complex modulus on shear strain (f=5 Hz); (**b**) dependency of complex viscosity and complex modulus with increasing frequency ($\gamma_{0}=10\%$); (**c**) dependency of storage and loss modulus on shear strain (f=5 Hz); (**d**) dependency of storage and loss modulus on frequency ($\gamma_{0}=10\%$).

Firstly, strain- and frequency-sweeps were performed for the neat epoxy resin to verify the Newtonian-like behavior. The complex viscosity of the system is independent of the applied strain amplitude and frequency, referring to Figure 4a,b, respectively. Storage and loss modulus show an independence of applied strain in strain-sweeps, as well (see Figure 4c). However, they increase with increasing frequency (see Figure 4d). In both tests, $G^{\prime \prime }$ is two magnitudes higher than $G^{\prime }$, revealing a fluid-like behavior of the resin. Therefore, taking Equation (8) into account following dependency is valid for the complex modulus:

(11) $$G^{*}=G^{\prime \prime }\;\mathrm{with}\;G^{\prime \prime }>>G^{\prime }$$

For a high MW polymer, a crossover in the frequency-sweep of the modulus occurs. This is not the case for the low MW epoxy resin. It can be considered as Newtonian-like fluid. The reciprocal value of the cross over frequency $\omega_{c}$ is considered as relaxation time λ.

(12) $$\lambda =\frac{1}{\omega_{c}}=\frac{1}{2\pi f_{c}}$$

### 4.2. Time-Dependent Behavior of Nanocomposite

**Figure 5:**
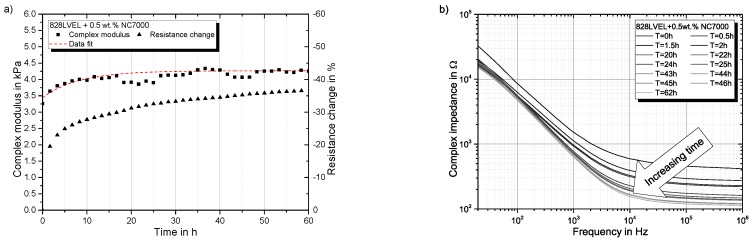
Change of (**a**) complex modulus and electrical resistance in oscillation and (**b**) complex impedance over time.

To verify the time-dependent behavior of the material, an oscillatory measurement with parallel DC measurement was performed; see Figure 5a. Beside that the change of AC electrical behavior was measured according to [44] to reveal the structural changes after the dispersion process; compare Figure 5b. Structural changes were highest in the first 10 h after dispersion. Therefore, the material was stored for 72 h before testing to achieve the steady state. The development of $G^{*}$ was fitted by a molecular growth model with the following formula [46] utilizing the Levenberg–Marquardt algorithm:

(13) $$y=A_{1}-A_{2}e^{-kx}$$

### 4.3. Strain Dependency of Nanocomposite

**Figure 6:**
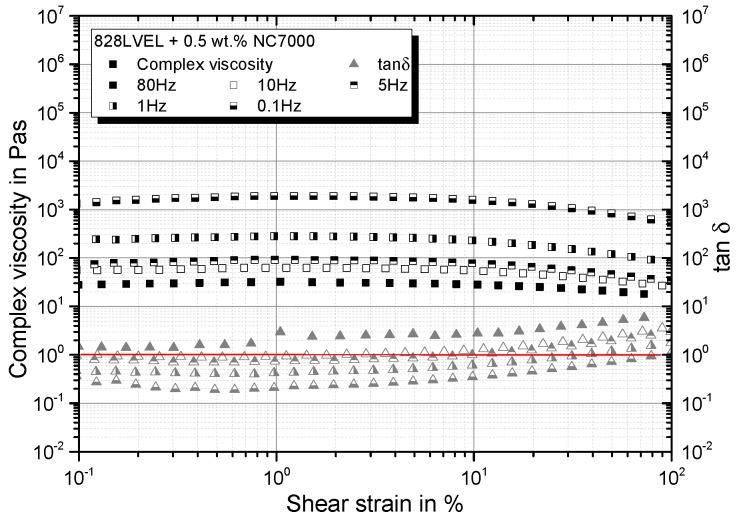
Shear strain dependency of complex viscosity and tanδ.

Going into detail for the strain-dependent rheological properties of the material, an initial plateau in the complex viscosity for all evaluated test frequencies occurs until a critical strain is reached. After that, distinct shear-thinning occurs; compare Figure 6. All curves of tanδ, except for 80 Hz, show a solid-liquid transition.

**Figure 7:**
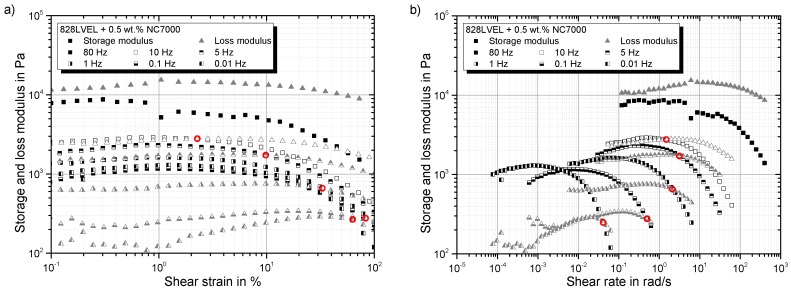
Dependency of storage and loss modulus on (**a**) shear strain and (**b**) shear rate.

In Figure 7a, the storage modulus $G^{\prime }$ and loss modulus $G^{\prime \prime }$ are plotted vs. the applied shear strain. The cross over, indicated by red circles, occurs for higher test frequencies at lower strains. This is considered as the yield point of the material, allowing the explanation of shear rate dependency. With Equation (6), the previous plot can be transferred into a shear rate $\dot{\gamma }$-dependent plot of storage and loss modulus; compare Figure 7b. The cross over occurs for higher frequencies at higher shear rate. Therefore, the structural change of the material seems to be shear rate independent. To reveal the structural changes in the material during the strain-sweeps, the electrical resistance was measured simultaneously.

For comparability, the measured resistance was normalized to the initial resistance. The resistance change can be calculated as follows:

(14) $$\Delta R=\frac{R-R_{0}}{R_{0}}$$

**Figure 8:**
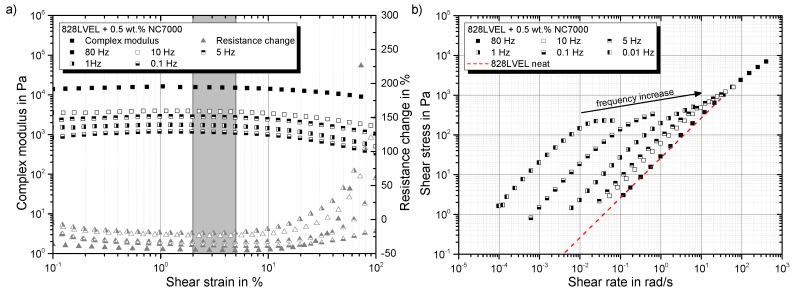
(**a**) Rheo-electrical material behavior in strain-sweeps at different frequencies; (**b**) shear stress in dependency of shear rate.

A negative value indicates an improved conductivity of the network structure in comparison to its initial state. In Figure 8a the complex modulus and the resistance change are plotted versus the applied strain. For low strains, the complex modulus increases and the resistance decreases. This indicates a more solidified particle structure. The minimum of the resistance change occurs in the same regime as the maximum in the complex modulus (indicated by the gray box in Figure 8a). With the beginning of the decrease of the complex modulus, an enormous increase in resistance change can be observed. This reveals the destruction of the conductive particle network. By utilizing Equation (7), the shear rate corresponding to the shear stress can be calculated. The log-log plot of shear stress vs. shear rate in Figure 8b shows the same slope as for the neat resin (red dotted curve) for all tested frequencies, but they are shifted. At a certain shear rate, just for low frequencies, the shear stress is limited and does not increase further by increasing shear rate. By reaching $\tau_{max,CNT}$, the destruction of the particle network begins, and consequently, shear-thinning occurs. For high frequencies, the material behaves more and more like the neat resin, and the yield point vanishes. Finally, for very high test frequencies, the material fully behaves like the neat resin, and the particles do not influence the rheological behavior despite the over percolated network structure.

### 4.4. Frequency Dependency of Nanocomposite

**Figure 9:**
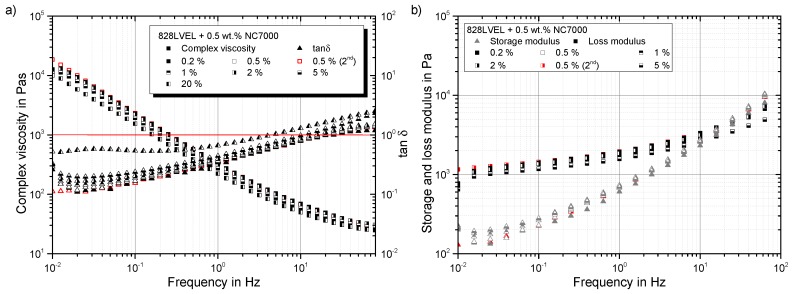
Frequency dependency of (**a**) complex viscosity and tanδ and (**b**) storage and loss modulus.

**Figure 10:**
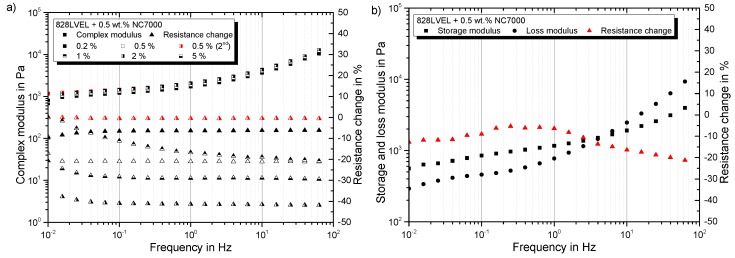
Frequency dependency of (**a**) complex modulus and resistance change and (**b**) storage, loss modulus and resistance change for a 20% strain amplitude.

The dependency of the complex viscosity in frequency-sweeps for different strain levels $\gamma_{0}$ is shown in Figure 9a. Additionally, the development of tanδ is given. For each frequency, an initial plateau in the complex viscosity is observed. This plateau disappears for a second performed frequency-sweep of the same sample (red symbols). All tested amplitude levels show a solid-liquid transition in the tanδ curves. This occurs for amplitudes over 5% at lower frequencies. Figure 9b gives solid-like behavior for frequencies below 11 Hz. The loss and storage modulus for 20% strain amplitude are plotted in Figure 10b.

Table 3 summarizes the relaxation times for the strain amplitudes. Higher amplitudes lead to higher relaxation times. This indicates a stronger network interaction.

The electrical resistance change and complex modulus in dependency of the frequency are plotted in Figure 10a. For all curves, the resistance decreases and ends up in a plateau after 1 Hz. Measuring the same sample a second time leads to no further change in resistance. This is shown exemplarily for a 0.5 wt.% filler concentration. The fact that with increasing frequency, no further changes in resistance occur leads to the assumption that the changes in the filler network structure are less dependent on frequency. For a strain amplitude of 20%, the resistance is constant and decreases further at frequencies of 1 Hz; compare Figure 10b.

**Figure 11:**
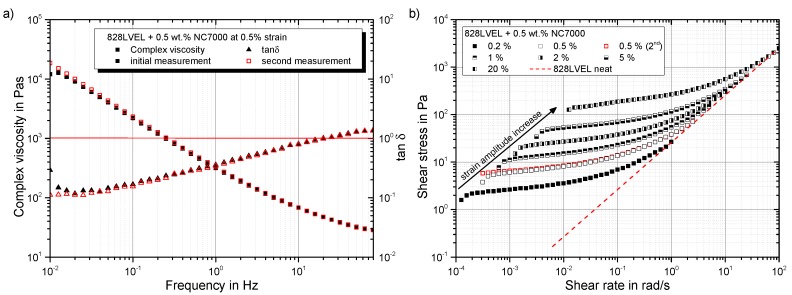
Dependency of (**a**) complex viscosity on frequency and (**b**) shear stress on shear rate.

The influence of a second measurement of the same sample in a frequency-sweep is given in Figure 11a. The initial plateau disappears, indicating no further changes in the network structure. Plotting shear stress vs. shear rate in Figure 11 shows an asymptotic convergence to the material behavior of the neat resin for high frequencies. Here again, the structural changes occur for low frequencies and therefore in the first oscillation cycles. At high frequencies, only the resin seems to be excited. A reasonable assumption is that the rigid particles are too inert to follow the movement of the oscillation. High frequency tests allow the measurement of the matrix viscosity without affecting the network structure.

Summarizing, the following observations were made:

(a) Low strain amplitudes in the strain-sweeps lead to a build-up of the filler network (decreased resistance)
(b) High strain amplitudes in the strain-sweeps destruct the filler network (exponential increase of resistance)
(c) The same material response for high frequencies in strain-sweeps
(d) Shear rate independent solid-liquid transition for the tested frequencies in strain-sweeps
(e) After initial resistance decrease, there is no further resistance change in frequency-sweeps
(f) Asymptotic convergence to the material behavior of the neat resin at high frequencies in frequency-sweeps

**Figure 12:**
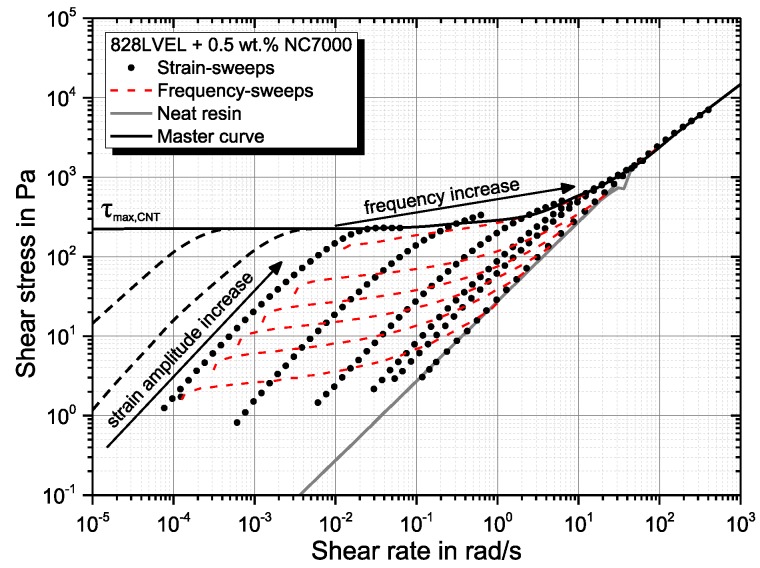
Master curve of the rheological properties of 0.5 wt.% NC7000 dispersed in Epikote 828LVEL.

**Figure 13:**
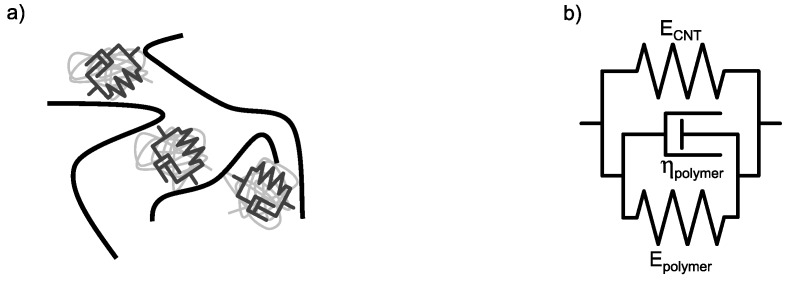
Schematical sketch of the (**a**) CNT-polymer-composite adapted from [23] and (**b**) the applied material model using mechanical elements.

Combining Figure 8b and Figure 11b leads to the master curve for the rheological material behavior of the nanocomposite shown in Figure 12. The black symbols indicate data gathered from strain-sweeps and red dashed lines the data from frequency-sweeps. The gray line indicates the material behavior of the neat resin. Increasing the shear amplitude leads to higher shear stresses resulting from a more pronounced solid behavior (high $G^{*}$) due to build-up of the particle network. The build-up is limited to a maximum strain. A further increase does not result in increasing shear stresses. The destruction of the particle network begins. The increase of the shear rate by an increase of frequency does not lead to a structural change. Therefore, no frequency-dependent increase of shear stress can be observed in the lower frequency range. With high frequencies, the material behavior is dominated by the neat resin. A schematic model is given in Figure 13a. The nanocomposite consists of a CNT and a polymer network. The CNT network is elastic and the polymer network viscous. Hence, the CNT network can be described by a spring with the modulus $E_{CNT}$ and the polymer by a classical Kelvin–Voigt element, consisting of a spring with the modulus $E_{polymer}$ and a dashpot with the viscosity $\eta_{polymer}$. A parallel circuit of both as shown in Figure 13b can be described with the equations given below.

The strain in all elements is equivalent.

$$\begin{array}{c} \epsilon =\epsilon_{CNT}=\epsilon_{polymer} \end{array}$$

The stress of the CNT with dependency on the applied strain ϵ is given by:

$$\begin{array}{cc} \sigma_{CNT} & =E_{CNT}\;\cdot \;\epsilon \end{array}$$

and the dependency of stresses in the polymer on applied strain ϵ and shear rate $\dot{\epsilon }$ is given by:

$$\begin{array}{cc} \sigma_{polymer,elastic} & =E_{polymer}\;\cdot \;\epsilon \\ \sigma_{polymer,viscous} & =\dot{\epsilon }\;\cdot \;\eta_{polymer} \end{array}$$

The total stress of the system results in the following equation:

$$\begin{array}{cc} \sigma_{tot} & =\sigma_{CNT}+\sigma_{polymer,elastic}+\sigma_{polymer,viscous}\\ & =E_{CNT}\;\cdot \;\epsilon +E_{polymer}\;\cdot \;\epsilon +\dot{\epsilon }\;\cdot \;\eta_{polymer} \end{array}$$

The stiffness of the CNT network is much higher compared to the polymer stiffness. This simplifies the equation as follows:

$$\begin{array}{cc} \sigma_{tot} & =E_{CNT}\;\cdot \;\epsilon +\dot{\epsilon }\;\cdot \;\eta_{polymer} \end{array}$$

with:

$$\begin{array}{cc} \lim_{\dot{\epsilon }\to \infty }\dot{\epsilon }\;\cdot \;\eta_{polymer} & =\infty \\ \lim_{\dot{\epsilon }\to 0}\dot{\epsilon }\;\cdot \;\eta_{polymer} & =0 \end{array}$$

and the constraint:

$$\begin{array}{c} \sigma_{CNT,max}=\tau_{max,CNT} \end{array}$$

the material responses as shown in Figure 12. At low frequencies, the CNT network dominates the material behavior. The stress scales with applied strain. For higher frequencies, the strain rate increases at constant strain, and therefore, the polymeric behavior becomes more dominant. For $\sigma_{CNT}>\tau_{max,CNT}$, shear-thinning occurs, resulting in no further increase of stress at low frequencies. By this observation, the superior efficiency of continuous dispersion processes like three-roll-milling to oscillatory methods like ultrasonication can be justified. The shear rate for ultrasonication is far higher, but the total amplitudes are lower compared to TRM. This study proved that high shear amplitudes are mandatory to separate agglomerates. Ultrasonication leads, despite the high energy level, not to the separation, but to the fracture of single rigid particles. The shear rate has a minor influence. The pretended shear-thinning in frequency-sweeps is not due to changes in the network structure, but rather corresponds to the rheological behavior of the polymer matrix. For steady shear flows, the absolute amplitude should be considered stronger than the shear rate.

## 5. Conclusions

Concluding, shear amplitudes are mandatory for changes is the CNT thermoset network structure. The shear rate has no influence on changing the CNT particle network, but the absolute shear strain. High frequencies do not change the network structure for low strain amplitudes in oscillatory rheology measurements. High test frequencies allow the determination of the rheological properties of the neat polymer matrix within the nanocomposite material.

## Figures and Tables

**Table 1:** Adapted process parameters for dispersion with three-roll-mill adapted from [44].

Step	Gap_Feeding_ μm	Gap_Dispersion_ μm	n_1_ Rpm	n_2_ Rpm	n_3_ Rpm
1	120	40	50	150	450
2	40	13	50	150	450
3–7	13	5	50	150	450

**Table 2:** Properties of Nanocyl NC7000 multi-walled carbon nanotubes.

Property	Unit	Value	Method of Measurement
Average diameter	nm	10	TEM
Length	μm	0.1–10	TEM
Carbon purity	%	90	TGA
Metal oxide (impurity)	%	10	TGA
Surface area	m${}^{2}$/g	250–300	BET

**Table 3:** Relaxation times λ for strain amplitudes $\gamma_{0}$.

Amplitude in %	$w_{c}$ in rad/s	$f_{c}$ in Hz	λ in s
0.2	150.80	24.00	0.041
0.5	125.35	19.95	0.050
0.5	125.35	0.2	0.050
1	114.35	18.20	0.055
2	95.13	15.14	0.066
5	68.86	10.96	0.091
20	27.39	4.36	0.229

Second frequency-sweep on the same sample.

## Abbreviations

The following abbreviations are used in this manuscript:

CNT	carbon nanotube
EP	epoxy resin
MW	molecular weight
MWCNT	multi-walled carbon nanotube
PC	polycarbonate
TRM	three-roll-mill
